# Supplementary material for: Loss of CDKN1A mRNA and Protein Expression Are Independent Predictors of Poor Outcome in Chromophobe Renal Cell Carcinoma Patients
Source: Cancers (Basel). 2020 Feb 17;12(2):465. doi: 10.3390/cancers12020465 (PMC7072616; doi:10.3390/cancers12020465)
Supplement: Supplementary file 1 [file cancers-12-00465-s001.pdf]

Supplementary Materials: Loss of CDKN1A mRNA and Protein Expression are Independent Predictors of Poor Outcome in Chromophobe Renal Cell Carcinoma Patients

Riuko Ohashi, Silvia Angori, Aashil A. Batavia, Niels J. Rupp, Yoichi Ajioka, Peter Schraml and Holger Moch

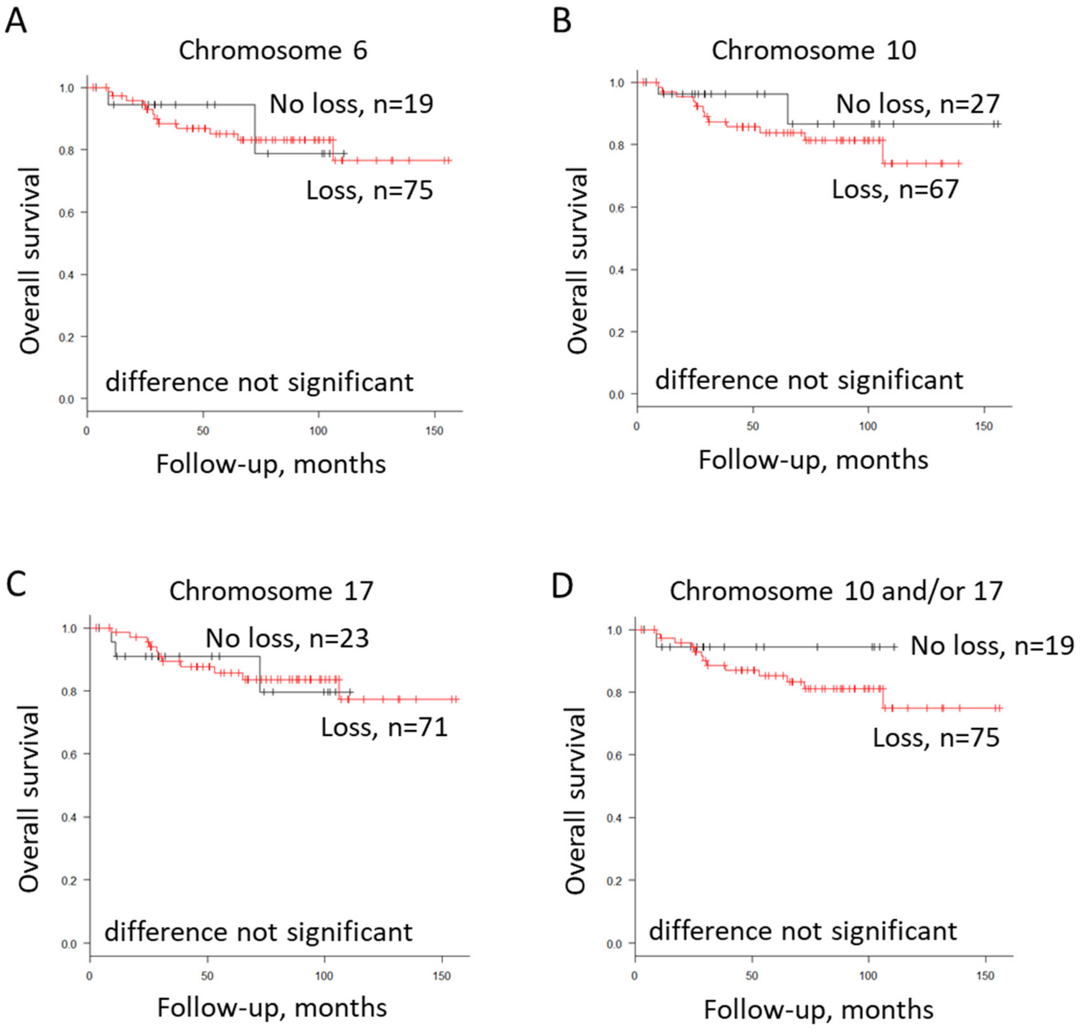

Figure S1. Loss of chromosome 6 harboring *CDKN1A* (**A**), chromosome 10 harboring *PTEN* (**B**), chromosome 17 harboring *TP53* (**C**), combined loss of chromosome 10 and 17 (**D**) and patient overall survival in chRCC.

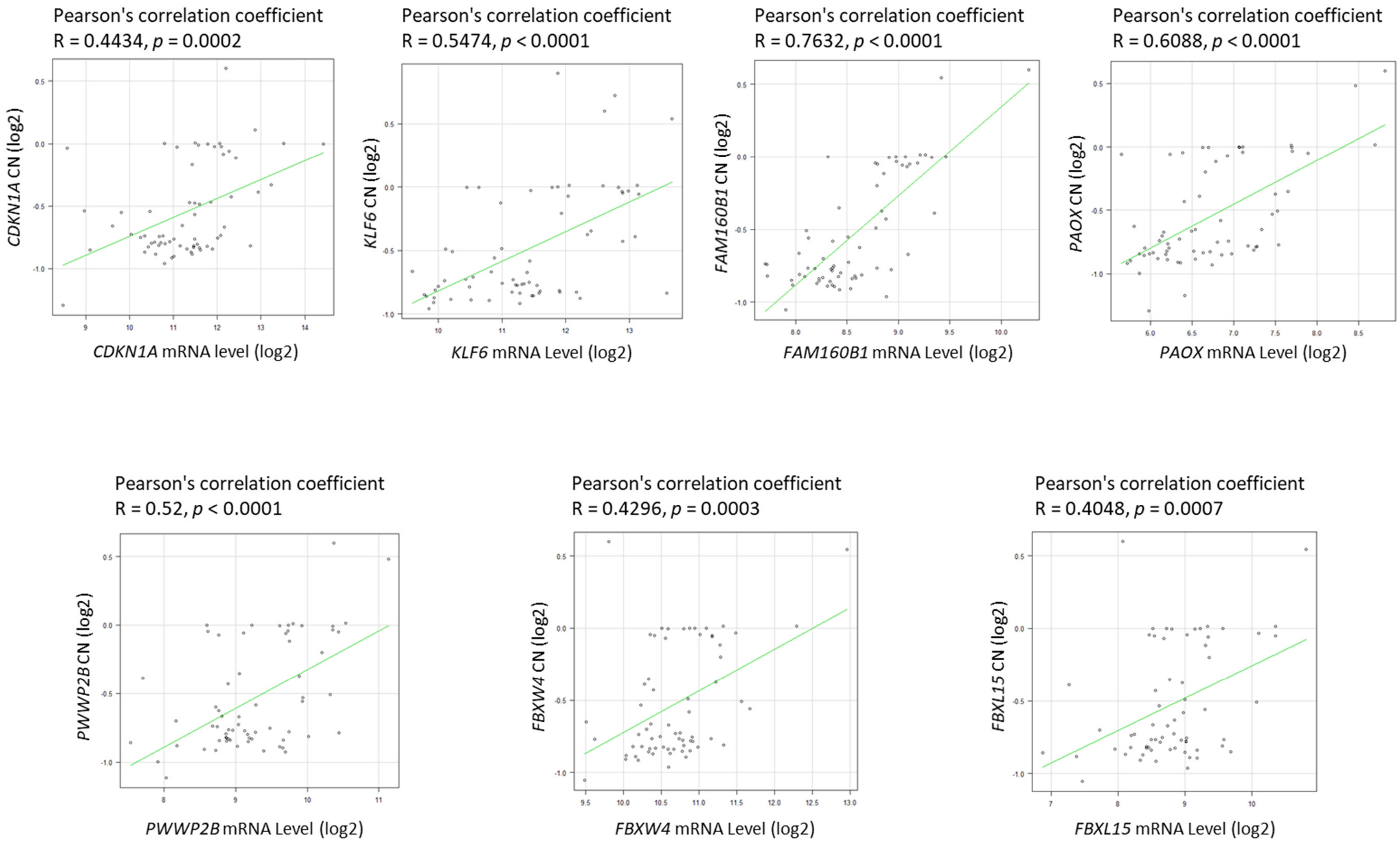
Pearson's correlation coefficient
R = 0.4434, *p* = 0.0002
CDKN1A CN (log2)
CDKN1A mRNA level (log2)
Pearson's correlation coefficient
R = 0.5474, *p* < 0.0001
KLF6 CN (log2)
KLF6 mRNA Level (log2)
Pearson's correlation coefficient
R = 0.7632, *p* < 0.0001
FAM160B1 CN (log2)
FAM160B1 mRNA Level (log2)
Pearson's correlation coefficient
R = 0.6088, *p* < 0.0001
PAOX CN (log2)
PAOX mRNA Level (log2)
Pearson's correlation coefficient
R = 0.52, *p* < 0.0001
PWWP2B CN (log2)
PWWP2B mRNA Level (log2)
Pearson's correlation coefficient
R = 0.4296, *p* = 0.0003
FBXW4 CN (log2)
FBXW4 mRNA Level (log2)
Pearson's correlation coefficient
R = 0.4048, *p* = 0.0007
FBXL15 CN (log2)
FBXL15 mRNA Level (log2)

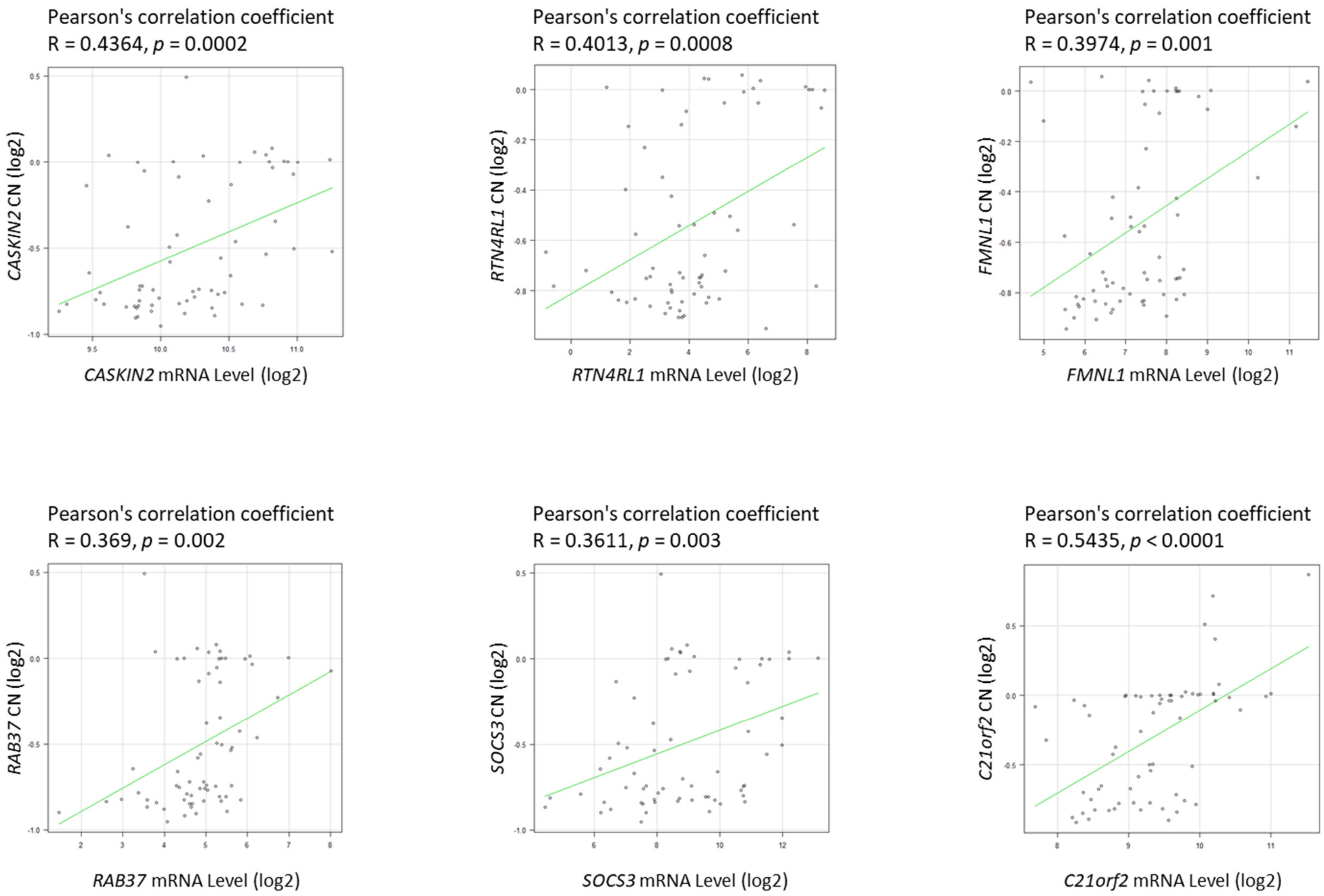

Figure S2. Correlation between CN loss and mRNA expression levels of 13 genes.

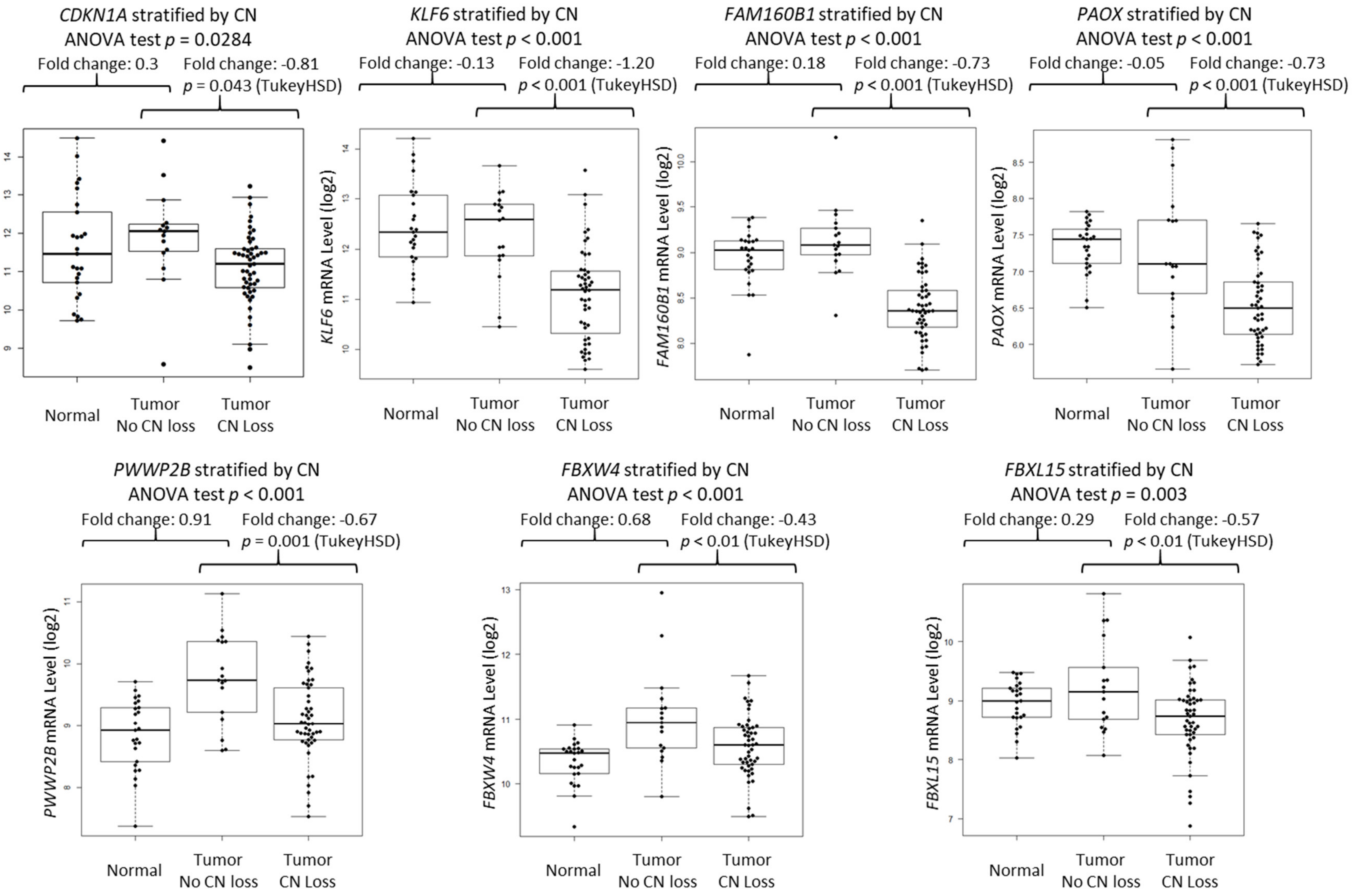
CDKN1A stratified by CN
ANOVA test p = 0.0284
Fold change: 0.3
Fold change: -0.81
p = 0.043 (TukeyHSD)
Normal
Tumor No CN loss
Tumor CN Loss
KLF6 stratified by CN
ANOVA test p < 0.001
Fold change: -0.13
Fold change: -1.20
p < 0.001 (TukeyHSD)
KLF6 mRNA Level (log2)
Normal
Tumor No CN loss
Tumor CN Loss
FAM160B1 stratified by CN
ANOVA test p < 0.001
Fold change: 0.18
Fold change: -0.73
p < 0.001 (TukeyHSD)
FAM160B1 mRNA Level (log2)
Normal
Tumor No CN loss
Tumor CN Loss
PAOX stratified by CN
ANOVA test p < 0.001
Fold change: -0.05
Fold change: -0.73
p < 0.001 (TukeyHSD)
PAOX mRNA Level (log2)
Normal
Tumor No CN loss
Tumor CN Loss
PWWP2B stratified by CN
ANOVA test p < 0.001
Fold change: 0.91
Fold change: -0.67
p = 0.001 (TukeyHSD)
PWWP2B mRNA Level (log2)
Normal
Tumor No CN loss
Tumor CN Loss
FBXW4 stratified by CN
ANOVA test p < 0.001
Fold change: 0.68
Fold change: -0.43
p < 0.01 (TukeyHSD)
FBXW4 mRNA Level (log2)
Normal
Tumor No CN loss
Tumor CN Loss
FBXL15 stratified by CN
ANOVA test p = 0.003
Fold change: 0.29
Fold change: -0.57
p < 0.01 (TukeyHSD)
FBXL15 mRNA Level (log2)
Normal
Tumor No CN loss
Tumor CN Loss

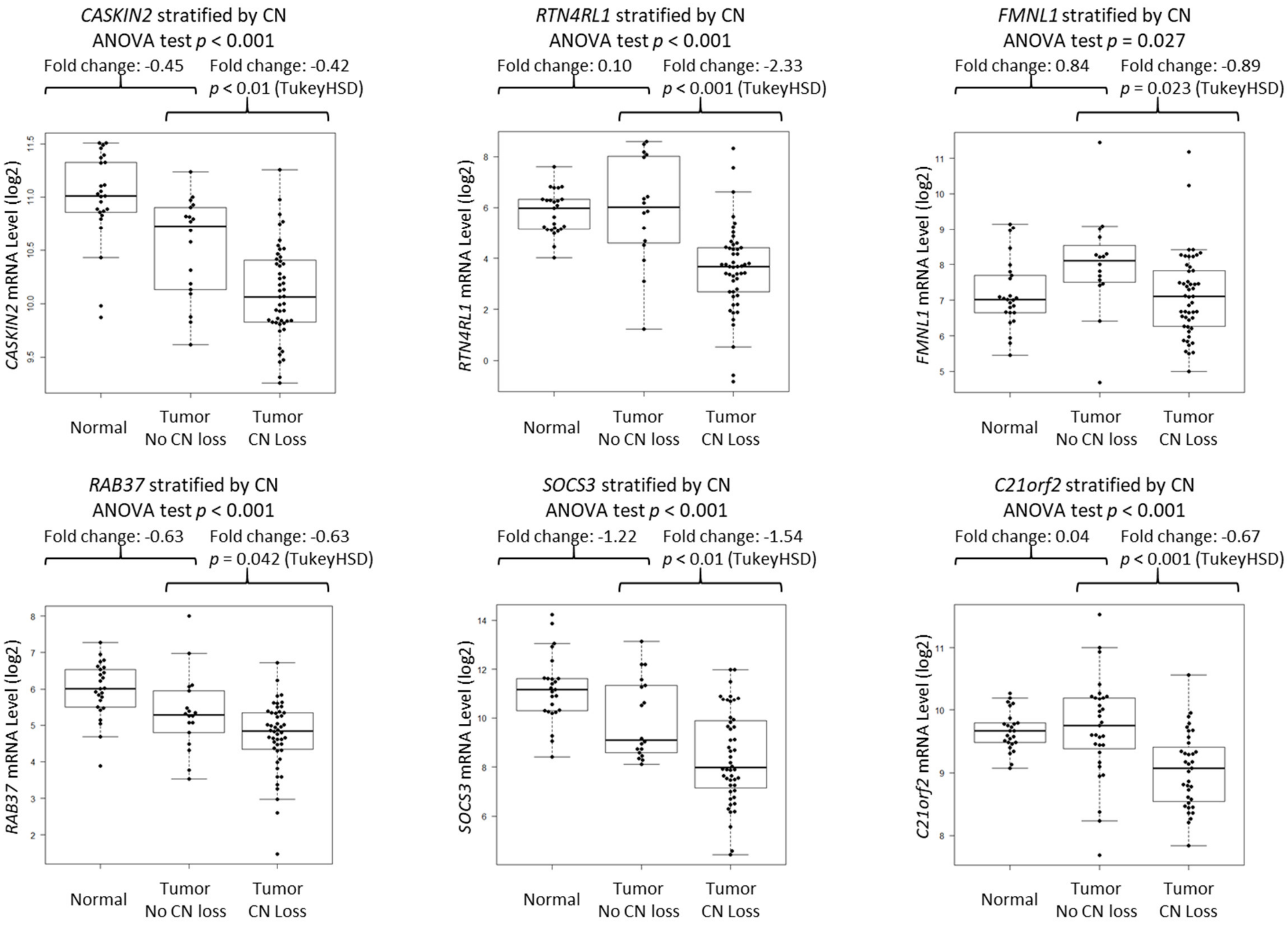

Figure S3. Scatter plots showing the correlation between mRNA expression and copy number variation of the 13 genes using the TCGA-KICH dataset. Dotted line: log2 threshold at −0.1 between CN loss and no loss.

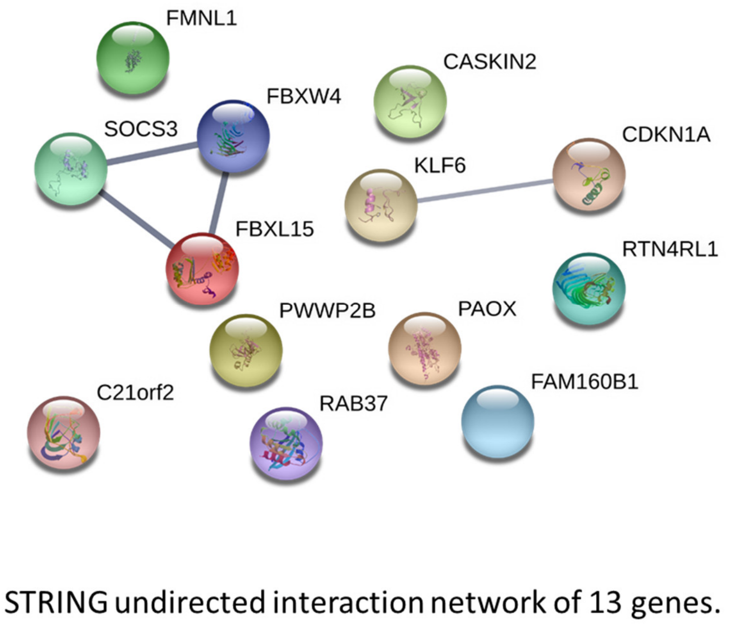

Figure S4. Protein-protein interactions between the 13 gene products using STRING database.

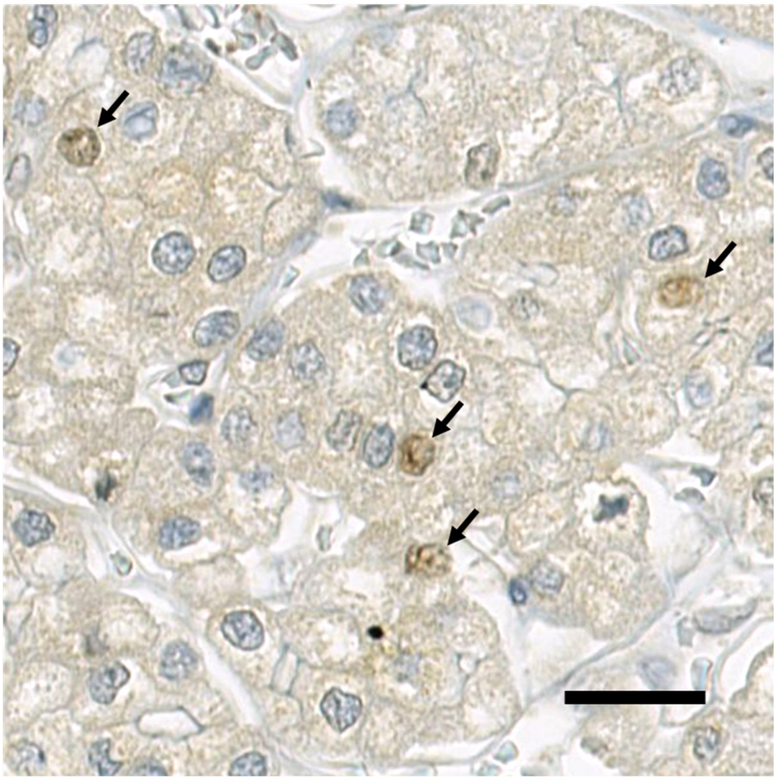

Figure S5. CDKN1A positive chRCC with weakly stained tumor cell nuclei (black arrows). Bar: 20 µm.

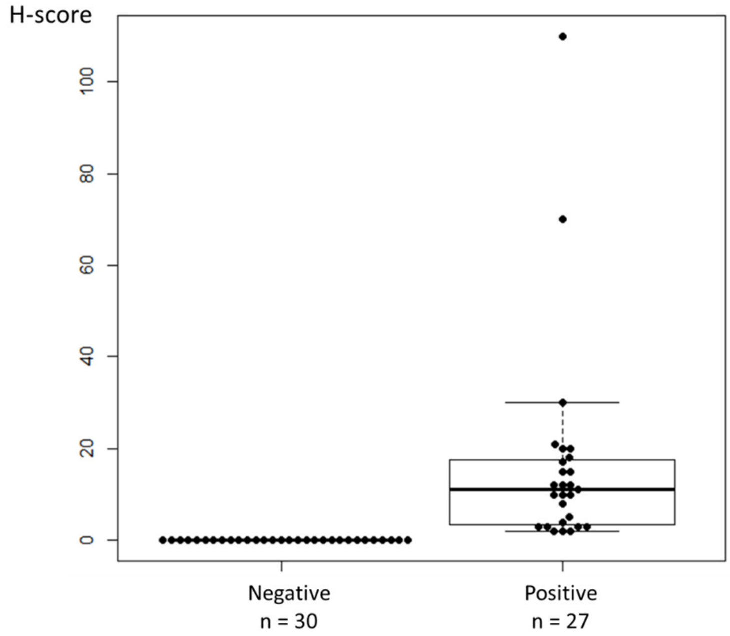

Figure S6. Distribution of CDKN1A H-scores of chRCCs by immunohistochemistry.

Table S1. Frequency of chromosomal loss in 2 chRCC cohorts.

**Cohort**	**Swiss cohort (** ***n*** **= 30)**	**TCGA-KICH (** ***n*** **= 64)**
Loss	*n* (%)	*n* (%)
Chromosome 2	22 (73.3)	47 (73.4)
Chromosome 6	25 (83.3)	50 (78.1)
Chromosome 10	19 (63.3)	48 (75.0)
Chromosome 13	21 (70.0)	43 (67.2)
Chromosome 17	23 (76.7)	48 (75.0)
Chromosome 21	17 (56.7)	33 (51.6)

Table S2. Genes with correlation of expression levels, median and best separation cutoffs, and survival (Data from Human Protein Atlas database).

Gene	Median Cut Off	Expression Level Range			Best Separation Cut Off	Expression Level Range		
		low (*N*)	high (*N*)	*p* value		Low (*N*)	High (*N*)	*p* value
CDKN1A	34.63	5.1–34.3 (32)	35.0–328.8 (32)	0.026	27.19	5.1–26.8 (25)	27.6–328.8 (39)	0.02
KLF6	18.64	5.2–18.6 (32)	18.7–101.7 (32)	n.s.	22.55	5.2–22.5 (40)	22.6–101.7 (24)	0.043
FAM160B1	2.5	1.1–2.5 (32)	2.6–9.2 (32)	n.s.	2.83	1.1–2.8 (40)	2.9–9.2 (24)	0.037
PAOX	1.86	1.0–1.8 (32)	1.9–9.7 (32)	n.s.	1.54	1.0–1.5 (19)	1.6–9.7 (45)	0.017
PWWP2B	9.26	3.6–8.9 (32)	9.3–35.2 (32)	n.s.	9.37	3.6–9.3 (34)	9.5–35.2 (30)	0.0039
FBXW4	14.06	6.6–13.9 (32)	14.1–66.8 (32)	0.021	13.23	6.6–13.1 (26)	13.4–66.8 (38)	0.0031
FBXL15	7.84	2.2–7.7 (32)	8.0–31.9 (32)	n.s.	5.95	2.2–5.9 (13)	6.0–31.9 (51)	0.0067
CASKIN2	8.23	4.7–8.2 (32)	8.6–21.4 (32)	n.s.	6.96	4.7–6.9 (17)	7.0–21.4 (47)	0.026
RTN4RL1	0.16	0.0–0.2 (32)	0.2–4.9 (32)	n.s.	0.08	0.0–0.1 (16)	0.1–4.9 (48)	0.015
FMNL1	1.06	0.1–1.0 (32)	1.1–18.5 (32)	n.s.	0.97	0.1–0.9 (34)	1.0–18.5 (30)	0.044
RAB37	0.25	0.0–0.2 (32)	0.2–1.7 (32)	n.s.	0.22	0.0–0.2 (26)	0.2–1.7 (38)	0.019
SOCS3	6.04	0.4–5.7 (32)	6.4–155.3 (32)	n.s.	6.6	0.4–6.5 (35)	6.7–155.3 (29)	0.031
C21orf2	3.88	1.2–3.8 (32)	3.9–15.2 (32)	n.s.	3.73	1.2–3.7 (29)	3.8–15.2 (35)	0.026

© 2020 by the authors. Licensee MDPI, Basel, Switzerland. This article is an open access article distributed under the terms and conditions of the Creative Commons Attribution (CC BY) license (http://creativecommons.org/licenses/by/4.0/).
